# Development of an Online Scenario-Based Tool to Enable Research Participation and Public Engagement in Cystic Fibrosis Newborn Screening: Mixed Methods Study

**DOI:** 10.2196/59686

**Published:** 2025-03-06

**Authors:** Louise Moody, Samantha Clarke, Matt Compton, Rachael Hughson-Gill, Felicity Boardman, Corinna Clark, Pru Holder, James R Bonham, Jane Chudleigh

**Affiliations:** 1 Centre for Arts, Memory and Communities Coventry University Coventry United Kingdom; 2 Warwick Medical School Warwick University Coventry United Kingdom; 3 Florence Nightingale Faculty of Nursing, Midwifery & Palliative Care King's College London London United Kingdom; 4 Pharmacy, Diagnostics and Genetics Sheffield Children's NHS Foundation Trust Sheffield United Kingdom

**Keywords:** extended genetic testing, next-generation sequencing, cystic fibrosis, decision-making, engagement

## Abstract

**Background:**

Newborn screening aims to identify babies affected by rare but serious genetic conditions. As technology advances, there is the potential to expand the newborn screening program following evaluation of the likely benefits and drawbacks. To inform these decisions, it is important to consider the family experience of screening and the views of the public. Engaging in public dialogue can be difficult. The conditions, screening processes, and associated moral and ethical considerations are complex.

**Objective:**

This study aims to develop a stand-alone online resource to enable a range of stakeholders to understand whether and how next-generation sequencing should be incorporated into the CF screening algorithm.

**Methods:**

Around 4 development workshops with policymakers, parents, and other stakeholders informed the design of an interactive activity, including the structure, content, and questions posed. Stakeholders were recruited to take part in the development workshops via purposeful and snowball sampling methods to achieve a diversity of views across roles and organizations, with email invitations sent to representative individuals with lived, clinical, and academic experience related to CF and screening. Ten stakeholders informed the development process including those with lived experience of CF (2/10, 20%), clinicians (2/10, 20%), and representatives from relevant government, charity, and research organizations (6/10, 60%). Vignettes constructed using interview data and translated into scripts were recorded to provide short films to represent and provoke consideration of families’ experiences. Participants were recruited (n=6, adults older than 18 years) to test the resulting resource. Study advertisements were circulated via physical posters and digital newsletters to recruit participants who self-identified as having a reading difficulty or having English as a second language.

**Results:**

An open access online resource, “Cystic Fibrosis Newborn Screening: You Decide,” was developed and usability and acceptability tested to provide the “user” (eg, a parent, the general public, or a health care professional) with an interactive scenario-based presentation of the potential outcomes of extended genetic testing, allowing them to visualize the impact on families. This included a learning workbook that explains key concepts and processes. The resulting tool facilitates public engagement with and understanding of complex genetic and screening concepts.

**Conclusions:**

Online resources such as the one developed during this work have the potential to help people form considered views and facilitate access to the perspectives of parents and the wider public on genetic testing. These may be otherwise difficult to obtain but are of importance to health care professionals and policymakers.

**Trial Registration:**

ClinicalTrials.gov NCT06299566; https://clinicaltrials.gov/study/NCT06299566

## Introduction

### Background

In the United Kingdom, every baby aged 5 days is offered newborn screening (the “heel-prick” test) for 10 rare but serious conditions [1,2]. The screening program aims to identify babies affected by genetic or congenital conditions before symptoms emerge in order to achieve the best outcomes through early treatment [1]. Screening in the United Kingdom is encouraged as a public health initiative [3], but it is an informed choice by parents who can decline it for their baby [1].

### Newborn Screening in the United Kingdom for Cystic Fibrosis

#### Overview

Each year in the United Kingdom, around 1 in every 200 babies will receive a positive newborn screening result for cystic fibrosis (CF) using first-tier biochemical testing. This result will initiate further diagnostic testing, including genetic testing, and around 250 will be found to have CF, 200 will be identified as “probable carriers” (which means they have one variant of the CF transmembrane conductance regulator gene responsible for CF), and approximately 25 children will receive an inconclusive outcome. This inconclusive outcome has been termed CF transmembrane conductance regulator (CFTR) related metabolic syndrome (CRMS) or CF screen positive, inconclusive diagnosis (CRMS or CFSPID) [4]. Children with CRMS or CFSPID have either a normal sweat chloride (<30 mmol/L) and two CFTR variants (at least one of which has unclear phenotypic consequences) or an intermediate sweat chloride value (30-59 mmol/L) and one or no CFTR variants [5,6]. Some of these children will go on to develop CF or a CFTR-related disorder, but most will remain well.

The current CF screening algorithm includes up to 50 of the most common gene variants associated with CF in the United Kingdom [7] and this detects most cases (about 97%) of CF. However, wider genetic testing of the CFTR gene would potentially allow more (several hundred) CF-causing CFTR gene variants to be identified [8,9]. Therefore, the use of extended genetic testing (next-generation sequencing [NGS]) is currently under consideration in the United Kingdom.

#### Potential Harms and Benefits of Incorporating Next-Generation Sequencing Into the CF Newborn Screening Algorithm

NGS could potentially increase the correct identification of CF (true positives) and therefore the number of children who would benefit from early treatment [10,11] and reduce the number of repeated bloodspot tests required compared with the current diagnostic pathway [12]. However, depending on how the testing is implemented, it could also have an impact on the number of inconclusive (CRMS or CFSPID) or missed results. Inconclusive results may lead to more diagnostic uncertainty; parents may be left unclear of how their child may be affected, and this may present interpretive dilemmas for clinicians [13]. A missed result is where the condition is missed through screening but later emerges through the presentation of symptoms (also termed a false negative) [14].

#### Specificity Versus Sensitivity

The United Kingdom National Screening Committee uses measures of “specificity” and “sensitivity” to help them decide how well screening works in a population [15]. Sensitivity refers to the test’s ability to correctly identify a baby with CF. A sensitive test will rarely miss babies with CF. Specificity is the test’s ability to correctly exclude a baby without CF. A highly specific test is more selective for variants that are known to cause CF, which means that there are few false positives (where babies are incorrectly thought to have the condition) or inconclusive results.

A specific approach to NGS for CF may mean missing a small number of babies with true CF (up to 10 per year in the United Kingdom; this includes those already missed [5 or 6 per year]). It would also reduce the number of babies given a designation of CRMS or CFSPID from 25 to around 5 per year. If a sensitive approach to NGS for CF were used, it might avoid missing additional babies with true CF but lead to the detection of more cases of CRMS or CFSPID (from 25 to 80 per year).

#### Decision-Making Around NGS

The parental experience of the screening process and receiving results is a particular concern for the development and operation of screening programs [16,17]. Parental confusion or anxiety about the implementation of NGS could lead to a reduction in newborn screening participation, resulting in treatable conditions going undetected. Parents need to have adequate information and understanding to consent to screening and understand the potential long-term implications of the results [10]. As well as the implications of positive results, the period of confirmatory testing following a positive screen can cause significant anxiety for the families as they wait for results [18,19] with potential impact upon family relationships, parental depression, and ongoing relationships with health care professionals (HCPs) [18-20]. The adoption of NGS could lead to knowledge that causes additional anxiety and has implications for the wider family’s health and reproductive decision-making [10]. Therefore, the use of NGS has prompted a range of concerns [21] and before the implementation of such advances, the impact on families should be considered [22]. Support from the public, and especially parents, is critical if extended genetic testing is to be successfully integrated into newborn screening [10].

Decision-making in the context of expanded screening and the use of genetic testing is complex. There are a range of considerations for policymakers weighing the advantages and disadvantages. Stakeholders engaging in the consideration of new screening programs have a range of technical, medical, legal, economic, ethical, psychological, and sociological concerns [21] to consider alongside the families’ experiences of screening, as well as the views of the public. Similarly, HCPs supporting the delivery of screening programs and interacting with parents as they reach a screening decision for their child also have complex information to relay and process. It is argued here that it is important to further explore and develop the ways in which screening information, including the benefits and potential disbenefits, is communicated to and understood by families and the wider public.

### Developing Online Tools

The use of online tools may offer a solution to relaying complex genetic information to families to aid their decision-making. There has been a global proliferation of digital health and online applications to address a range of health-related needs including training and education, condition management, health care records, disease screening, diagnosis, and monitoring [23]. As well as technology designed for specific conditions and condition management, users expect to access health information online to inform their understanding and decision-making, predict a prognosis, and cope with illness [24-26]. Parents are no different in their use of the internet to search for information about their child’s health and guide their health-related decisions [26]. There are limited online tools related to newborn screening, with the most comprehensive and reliable sources being those provided by the National Health Service (NHS) to support parental decision-making about screening for their child, rather than considering wider policy questions.

The research team has led and delivered a range of research projects exploring parental experiences of newborn screening, as well as research considering stakeholder perspectives on the potential expansion of screening programs [27-37]. We have found that due to the nature of the inherited conditions and the complexity of the screening process, communicating the potential outcomes of screening and their implications during the research process, consultation, and public engagement activity is challenging [38]. However, within the context of newborn screening, without end-user engagement, we may constrain the desired outcomes of the screening programs as well as the information sources developed to support them [39-41].

Understanding the benefits and potential disbenefits of different approaches to screening can be complex for several reasons. The way screening programs are evaluated is complex and involves measuring concepts some stakeholders are unlikely to have engaged with before. Also, the conditions screened for are rare, meaning the general public may not have heard of them. This makes them less likely to engage in research or stakeholder engagement around them [42,43]. Finally, newborn screening consent processes are often less than desirable and not recognized as a choice [44], which can mean the general public does not see the relevance or engage in research around it.

It is argued here that to make the information accessible and understandable, there are elements and techniques from storytelling and aspects of game design that can be applied. For example, scenario-based approaches and storytelling, and encouraging game-like behaviors (such as interaction and learning) in order to build engagement and motivate the user [45,46]. A previous project demonstrated the difficulties of engaging the public with research exploring the views and experiences of people with genetic conditions and highlighted the need for innovation and creativity in this area [47]. The approach taken here seeks to develop knowledge, facilitate critical thinking, and build empathy with the experiences of families, as well as interest and confidence in complex concepts and scenarios [48-51]. The study, therefore, adopted a game-based intervention development process [52] and a storytelling approach using scenario-based narratives [51] to encourage interaction and sufficient understanding to inform decision-making.

### Goal of the Work

We aimed to consider a new approach to engage and consult with stakeholders. We sought to develop a stand-alone resource to enable a range of stakeholders to understand and consider the question “How should NGS be incorporated into the CF screening algorithm?”

## Methods

### Overview

We sought to develop an online tool to facilitate clinical and stakeholder consultations related to newborn screening. To develop an effective tool, an iterative user-centered development process was adopted, informed by principles from games research and interdisciplinary approaches to building an online narrative interaction [51]. User-centered design draws on research and understanding across a range of disciplines to center the design of innovation (eg, products, software systems, educational resources, service delivery, and so on) around the knowledge and understanding of those that will use it, in order to optimize ease of use, effectiveness, efficiency, and satisfaction [53-55]. The development of the tool was informed by collaboration with a range of stakeholders and built upon previous research undertaken with parents and HCPs [27,38,56,57].

### Recruitment

#### Stakeholder Group

Stakeholders were recruited via purposeful and snowball sampling methods to achieve a diversity of views across roles and organizations. Email invitations were sent to representatives from the European CF Society, newborn screening laboratories, NHS England, consultant pediatricians specializing in CF, the NHS Newborn Blood Spot Screening Program, Genomics England, CF Clinical Nurse Specialists, the Cystic Fibrosis Trust, individuals with lived experience of CF either personally or as a parent, and academic experts in newborn bloodspot screening (NBS) and medical ethics. This approach ensured that the development of the tool was informed by both direct and indirect knowledge of a range of different family experiences of NBS. Members formed an oversight group that provided input and feedback on development.

#### Testers as Potential Users

In addition to the stakeholder groups, participants were recruited to test the resulting tool. Study advertisements were circulated via physical posters and digital newsletters, as well as via a social enterprise and Coventry University support structures for academic writing and English as a second language. Participants were offered a US $25 shopping voucher to thank them for their time.

#### Iterative Codevelopment of the Online Scenario-Based Tool

The stages through which stakeholders were involved in the codevelopment of the tool are given in Table 1. Initially, concept development workshops were undertaken to scope out the purpose of the tool and the requirements of the various stakeholders. This was followed by the development of filmed scenarios, written content within an interactive workbook, and an online tool. These were further developed and refined based on feedback from stakeholders and the group of user testers.

**Table 1:** Stages of stakeholder involvement and codevelopment.

Stages of development	Roles and involvement in the development process	Purpose of the development activities
About 2 concept development workshops	Research team plus stakeholder group members from the CF Trust Research team with 6 members of the stakeholder group (3 from NHS England, 1 pediatrician, and 1 pediatric nurse)	Determine the scope of the system and decisions to enable via the system Define stakeholder requirements for the system Highlight any challenging concepts that may need support with additional information
Initial ideas and content development	Research team activity	Based on the scope defined in the workshops, the academic team selected suitable interviews to illustrate the scenarios and form vignettes
Development of the site structure	Research and technical team activity Workshop session with academic team	Refine system requirements Develop the structure of the system on paper Test structure with the research team
Script and workbook development	Research and video production team	Iteratively developed vignettes into scripts Develop supporting workbook content to provide additional information Review and revise the full draft of scripts and workbook by the research and production team Further drafts reviewed through 1:1 meetings with pediatric nurses and meetings with National Health Service - England
Script and website structure review	Research team and stakeholder group (2 National Health Service - England, pediatric consultant, pediatric nurses, and 3 academic specialists)	Review the script in advance of a facilitated workshop session to identify issues, refine the messaging, and add contextual details
Script and workbook finalized and signed off	The research and production team revised the script based on the feedback Script signed off by the stakeholder group	Final script reviewed by a wider stakeholder group by email and agreement sought that filming could commence
Filming	Research and production team A health care professional (child nurse) was present to guide the accuracy of the clinical experience and interactions	Actors receive the scripts Around 4 days before recording, a read through was held via an online meeting The scenes were recorded with professional actors in health and home simulation facilities
Film production	Production team	Films were recorded, edited, and produced Films were edited following feedback from the research team
Development of the digital tool and interactive activity	Technical team	Creation of structure of the digital tool within WordPress Developed interactive workbook Several iterations based on feedback from the research team to improve structure and usability Test sheets logged the usability issues and agreed actions to resolve
Oversight group review of the films	Workshop with stakeholder group	Review of the videos to ensure clinical accuracy and appropriate representation within an NHS context via a workshop Revisions to the videos based on the feedback Videos inserted into the digital tool
Final review of the digital tool and interactive activity	Stakeholder consultation	Stakeholder group reviewed the digital tool, particularly the questions being asked via the polls or survey element
Launch of the digital tool	Technical team	Digital tool and interactive activity made available as open access
Review of digital tool accessibility	Research team with testers as potential users	Readability and acceptability testing by potential users to improve accessibility

#### Development Workshops

As outlined in Table 1, a series of 4 workshops were undertaken to inform the iterative development. The number of participants varied per workshop, but across the 4 workshops, there was representation from the European CF Society, newborn screening laboratories, NHS England, a consultant pediatrician specializing in CF, NHS Newborn Blood Spot Screening Program, Genomics England, a Clinical Nurse Specialist, the Cystic Fibrosis Trust, individuals with lived experience of CF either personally or as a parent, and academic experts in NBS and medical ethics. The workshops aimed to ensure that the tool remained focused on the key issues and questions we wished to ask, provided suitable messaging, represented NHS best practices, and also reflected parents’ actual experiences with newborn screening.

#### Development of the Tool

Acknowledging the development challenges of creating an effective digital tool, production guidance was applied from the transdisciplinary methodology of game-based intervention design [52], and the development process was managed over 3 cycles: preproduction, production, and postproduction. Technical development quality considerations were observed from the standards outlined in the CISQ Quality Characteristic Measures of Software Coding Standards [58]. As outlined in Table 1, the structure of the digital tool and interactive workbook was initially developed by the technical team and iterated based on feedback from the research team. The stakeholder group tested and provided feedback on the individual elements (eg, videos and other interactive elements) during both workshop sessions and 1:1 reviews. They also provided a final review and approved the digital tool and interactive activity.

#### Usability and Acceptability Testing

The final prototype was usability and acceptability tested via walkthroughs of the tool. Data collection involved either an online or face-to-face session that lasted between one and two hours. Participants walked through the website at their own pace and navigated through it “naturally.” After exploring each page, participants were encouraged to give both positive and critical feedback. They were guided by a series of usability and readability prompts based on readability assessment tools (Suitability Assessment of Materials, Comprehensibility Assessment of Materials [59], the Health Literacy Index [60], and key usability principles [61]). The sessions were recorded (video and audio) and transcribed.

### Ethical Considerations

The research was approved by the Coventry University Ethics Committee (P149430 and P133880). All participants consented to their involvement. Data were pseudonymised. Participants did not receive compensation for their involvement.

## Results

### Sample

A total of 10 (N) stakeholders took part in the development process, including those with lived experience of CF (2/10, 20%), clinicians (2/10, 20%), and representatives from relevant government, charity, and research organizations (6/10, 60%). Everyone that was approached agreed to take part.

A total of 16 people responded to the call for participation, who self-identified as having a reading difficulty or having English as a second language to test the resulting tool. Among them, 9 adults either dropped out or did not respond to follow-up emails, and 1 did not meet the inclusion criteria as they had significant previous knowledge or experience of CF. In the end, 6 adults (older than 18 years of age) were recruited to test the resulting tool.

### The Concept and Focus

The development process enabled the definition of an online tool that would (1) explain to the general public 2 different ways NGS could be incorporated into the CF screening algorithm in the future (sensitive or specific approaches), (2) allow us to collect public and stakeholder views on these 2 different ways of implementing NGS to inform policy decisions and research, and (3) demonstrate that the public can engage and contribute to very specific and complex issues in health care when given appropriate information and tools.

The 4 development workshops enabled the exploration of the implications of NGS [38,56,57]. It was decided that the interactive tool would focus on the question: “How should NGS be used when screening babies for cystic fibrosis?”

It was agreed that the online format would enable wider and more geographically distributed public views to be considered. In previous research, the team developed short PowerPoint presentations to explain newborn screening concepts to participants and collected views through interviews and workshops [38,56,57]. An online tool would enable the team to explain complex concepts more effectively and potentially enable data collection on a larger scale.

The tool focused on understanding public views on whether a “sensitive” or “specific” approach should be adopted if NGS were to be incorporated into the CF screening algorithm. An outcome of the workshops (and informed by the games-based approach) was the decision that the potential impact of the 2 different approaches (sensitive and specific) would be explored through the use of video-based storytelling to bring the concepts to life and build empathy with family experiences.

Having established the potential implications of the specific and sensitive tests we sought to represent and tell the family experience through 4 scenarios:

- Scenario 1: A “not suspected” or “normal screening result”: In this scenario, it is unlikely that the baby has CF. The screening outcome is normal and no additional follow-up is required. The vast majority of babies will have a “not suspected” or “normal newborn screening result” and these families will be notified about their baby’s normal test results by 6 weeks of age.
- Scenario 2: CFSPID: Sometimes, newborn screening results suggest that a baby could have CF, but the baby is healthy and follow-up tests do not confirm CF but rather indicate an inconclusive sweat test result and the baby is described by a designation CRMS or CFSPID. Most children with CRMS or CFSPID will remain well, and their health will not be affected by this result, while a small number may go on to develop CF or a CF-related disorder.
- Scenario 3: Missed CF: Babies with a normal NBS result sometimes turn out to have CF. This is known as a “false negative” or “missed” CF result. These cases are usually identified after a baby or child presents with physical symptoms of the condition and further investigations are carried out. All screening programs can produce false negative results, although efforts are made to minimize them and ensure babies are identified and treated as soon as possible.
- Scenario 4: True positive, CF confirmed: A small number of babies will have a positive screening result for CF (about 1 in every 3000 babies screened). These results are communicated to parents by a specialist HCP within a few days of becoming available so that the baby can be assessed quickly and, if needed, start treatment. Follow-up tests (such as a sweat test) will be performed to determine if the baby has CF.

### Representing the Family Experience

To ensure accurate representation of family experiences, it was agreed to use anonymized data previously collected from parents about their screening experiences [27,62]. Vignettes were constructed using interview data based on interviews with 16 participants (parents) who had experienced a positive CF NBS result; 6 were parents to a child with CF, 3 were carrier parents, and 7 were parents to a child with CFSPID to represent each different scenario. We also sought to show varying emotions over time as the diagnoses unfolded for families. These were then formed into scripts by the research team guided by a producer. The interview transcripts were iteratively developed by the research team and the media producer into production scripts. We brought together stakeholders with different perspectives (ie, from different roles and organizations) who have worked with families with a wide range of experiences to inform the development of the scenarios. Stakeholder feedback was sought after each iteration, and this led to changes that ensured accuracy in terms of the screening pathway and clinical information as well as portraying an authentic parent experience in the media content.

### Filming and Production

Once approved the scripts were translated into a production plan for the 4 scenarios, and research into location, casting, costume, and clinical props was undertaken. The main roles for each film were cast through a talent management agency. Actors’ profiles were screened and selected in light of their past acting experience as well as their age and image for their suitability within each role. The actors playing the parental roles were selected in line with our interview sample and data [27] and the 2023 UK CF Registry Annual Data Report [63], which indicates only 5.4% of the UK CF population are of non-White or mixed ethnicity. Diversity of representation was considered through the casting of non-White actors to portray HCP roles and variation in the presented family dynamics (eg, inclusion of an older father, regional accents, and a single-parent family). Additional clinical roles with little to no dialogue were assigned to stakeholders, colleagues, and crew due to budget limitations.

Costumes and the relevant clinical props were sourced through the lead university and from clinical stakeholders. The locations for the filming were chosen to not only provide a suitable range of clinical settings that would reflect those used throughout the screening process but also to cater to each family’s home setting. The Faculty of Health and Life Sciences Facility at Coventry University incorporates a range of simulation facilities including hospital wards and consulting rooms, as well as 2 mock houses built for student training that could be repurposed for each of the family homes.

The 4 scenarios were filmed within a 3-day period to meet constraints around actor, stakeholder (on set as advisors), and location availability. This approach required 2 film crews totaling 8 production operatives to work in parallel during the first day of production and a single film crew of 4 production operatives on the second day. The filmed scenarios were edited into a reflective narrative for each short film. Many hours of filmed content were reduced into short narrative dialogues of no more than 5 minutes in length to allow for online delivery within the interactive activity.

During the rough-cut stages of postproduction, the initially edited sequences were reviewed by the stakeholder community and were assessed based on the realism of the actor’s delivery, focusing on their emotional journey as well as the clinical accuracy portrayed. Several iterations were produced and reviewed during the processes until the content was approved for use in the interactive activity, at which point a final cut was produced for each of the 3 films where the audio was enhanced and the images were color graded to reflect the emotion of each parent’s journey through newborn screening.

In parallel to the production of the films, the development of the online digital tool commenced.

### Preproduction Considerations

The hosting service “Domain of One’s Own” [52,58,64] was chosen as a cost-effective and easily accessible web hosting platform with access to more than 100 open-source applications. WordPress [65] was chosen from the open-source applications as it provides a number of built-in tools and features, such as prebuilt website themes, infrastructure security, automatic backup, and a large catalog of free-to-use plugins for customization and user-experience design. The Elementor (Elementor Ltd) plugin, for example, supports a “drag and drop” responsive approach to creating and editing websites. This add-on supported customization of the website layout, theme, and structure, and minimized development time. With minimum coding required, the development team could quickly build and test content sections to test the user journey and flow through the website. This supported an iterative design and development cycle, in which both the website infrastructure and delivery of the content could be modified quickly. Due to the complexity of the proposed content, it could be packaged into sections and appointed pages, allowing the user autonomy in deciding what content and information was relevant to their needs.

### Production Considerations

An architectural map of the website structure was codeveloped with the stakeholder group. The mapping activity aided an analog approach to planning the user’s interaction and experience. It helped prioritize content, which was ordered into either essential or supplementary information, informing the design, layout, naming, and signposting within the website’s structure.

#### Layout and Content

**Figure 1:**
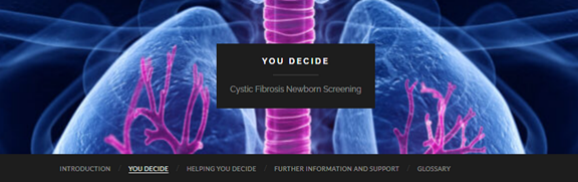
Cystic fibrosis newborn screening: you decide site structure.

The layout of the online tool is available on the CF Newborn Screening: You Decide website [66]. It is comprised of 5 sections as given in Figure 1. An introduction section explains the purpose of the site. The “You Decide” section contains the question for the user to consider alongside the 4 filmed experience scenarios, as well as a survey link enabling the capture of the user’s view on the question: “How should NGS be used when screening newborn babies for cystic fibrosis?” An interactive workbook is provided on the “Helping You Decide” page. It is recommended that the user reads the information and plays through all of the videos before sharing their views via the survey link. The activity takes approximately 40 minutes to complete.

#### Presentation of Filmed Scenarios

Using the Elementor plugin, each video scenario was laid out in an order to view. The video scenarios were labeled and displayed using a visual template to show the viewing order and progression to the next scenario. Audio, caption support, and control features (such as pause, fast forward, backward, skip, and replay) were added to each video playback template for user access and control over the information being presented.

#### Interactive Workbook

**Figure 2:**
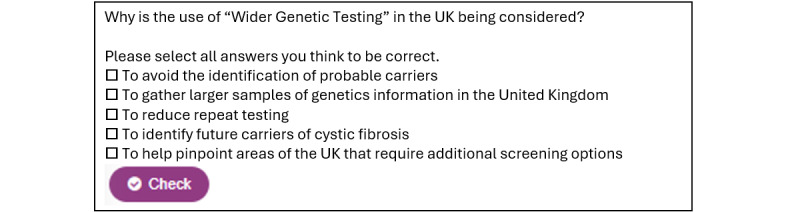
Example of a question to encourage user engagement with the workbook content.

During the development, stakeholders agreed that, as well as the filmed scenarios, further information would be beneficial for users. The resulting “Helping You Decide” section contains background information about CF, newborn screening, screening test outcomes, genetic testing, and specific versus sensitive tests. The user is encouraged to familiarize themselves with the interactive workbook content to enable an informed decision, but it is possible to skip through the sections depending on what the user may already know or choose to explore. There is also a glossary of key terms for reference. The interactive workbook was developed using the HTML 5 package plugin to present information in selectable and skippable sections. To encourage user engagement and interaction with the workbook content, gamified interactive elements were used, including multiple-choice quiz formats, memory games, flashcards, drag-and-drop elements, and interactive images (Figure 2).

#### Capturing Views

With the aim of facilitating public engagement and capturing their views, polls and a survey were embedded within the tool.

**Figure 3:**
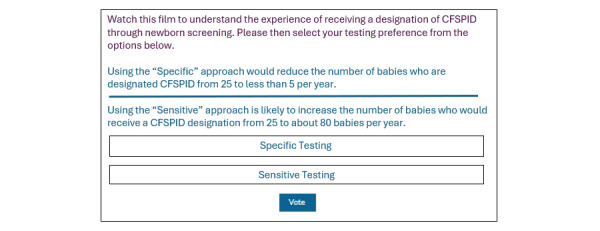
Example of a question to prompt immediate reflections after watching a filmed scenario. CFSPID: cystic fibrosis screen positive, inconclusive diagnosis.

As the user works through the filmed scenarios, they are asked to complete the polls prompting their immediate responses to each of the filmed scenarios (Figure 3). The “Poll Maker” plugin was embedded to create these online polls. It was recognized the user’s view may change as they go through the experiences, and assimilate more information.

**Figure 4:**
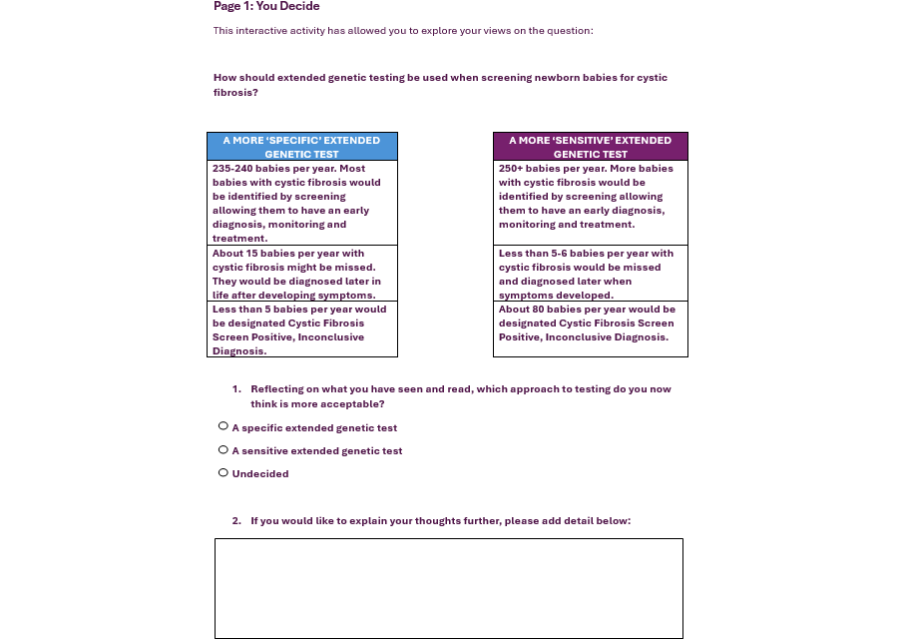
Question to gather a final view of the user on the question: “How should extended genetic testing be used when screening newborn babies for cystic fibrosis?” CFSPID: cystic fibrosis screen positive, inconclusive diagnosis.

Once the user has watched all 4 experiences, they are asked to share their final decision via the “My Decision” survey (Figure 4). This final decision question is situated within an online survey software (JISC Online Surveys). Currently, the polls, surveys, and interactive elements are anonymous and do not collect any identifying data from those responding, but the use of online survey software enables the addition of informed consent processes, if required, for data retention, analysis, and use, as well as the collection of additional demographic information (if required).

#### Thematic Design

**Figure 5:**
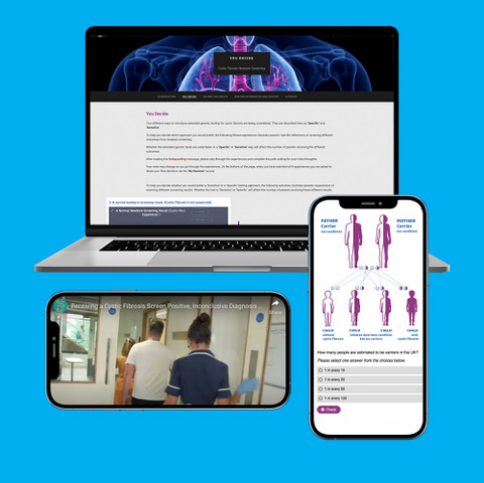
Thematic style.

The color stylization of the website (Figure 5) was designed to match the purple CF awareness ribbon and the NHS blue logo to reinforce end-user recognition, acceptance, and clinical validity of the website content. Presentation of text was standardized to aid visual identification of links to information sources as well as key information or terminology. Images used were either under a Creative Commons license or purchased with an educational use license.

#### Safeguarding

Due to the potentially sensitive nature of the content (eg, experiences of receiving a diagnosis of a long-term condition and discussions of reproductive outcomes), it was agreed among the stakeholders that a safeguarding message should be displayed. Further guidance and sources of urgent and nonurgent support were also signposted.

### Postproduction Considerations

#### Overview

As part of the iterative design and development process, the tool was tested by users. A “test sheet” template was first created to guide the stakeholder group on how to log technical flaws, and editing needs, and highlight areas for reassessment. The site went through 3 iterations of testing with stakeholders before being tested with new users.

#### Usability and Acceptability

Usability and acceptability were tested through walkthroughs by 6 novice users. They found the tool easy to use and did not struggle when interacting with or navigating the site or the interactive workbook. Participants liked the colors and design of the site, feeling that it conveyed the right tone.

They reported that the videos were engaging, elicited empathy, and helped to form an understanding of the parents’ experiences. One participant commented, “It gets more interesting, I just want to keep going on and on.”

They felt the videos elicited empathy for the parents and helped build their experiences of the test results*.* Several of the participants commented on how the emotional storytelling and representation of the family experience helped them to understand that “experience is the best teacher” and “…you learn more through people’s experience. That’s the fact of life.”

There were some usability issues identified with the videos, specifically their size on different devices and the number of interactions or clicks needed to access and progress through the videos. The interactive workbook element was mostly considered easy to understand, and it helped users to form an understanding of the differences between the specific and sensitive approaches to testing. One participant noted, “It gives me a lot of information about this, which I really like.”

Multiple participants stated they liked the engagement and interactive elements, specifically that the questions helped their understanding by drawing attention to the main points and encouraging them to reread if they had not understood.

To further improve readability, participants suggested reducing the amount of text, shortening the length of individual pages, adding a read-aloud function, and supplementing text with additional images and diagrams. One participant shared, “I love graphics. I love pictures, so I’m seeing this will give me more interest to go through it.”

They found the pictures and diagrams to be an engaging and accessible way to summarize information, drawing attention to a comparison table graphic that helped them to understand the difference between “sensitive” and “specific” testing.

## Discussion

### Principal Findings

The “CF Newborn Screening: You Decide” tool was conceived as a novel approach to engage the public and stakeholders in addressing the complex issues and debates around newborn screening. Through an iterative design process, in collaboration with key policymakers (eg, NHS England) and stakeholders (eg, parents and clinicians), a stand-alone resource has been developed to enable the public to understand and consider the question: How should NGS be used when screening babies for cystic fibrosis? It is intended that the tool will help people to form considered views and facilitate access to the perspectives of parents and the wider public on genetic testing that are otherwise difficult to obtain but are of importance to HCPs and policymakers.

As an open-access online resource, the “user” (eg, a parent, a member of the general public, or an HCP) is provided with an interactive presentation of the potential outcomes of NGS, allowing them to visualize the impact upon families through storytelling. The initial feedback suggests that the stories or filmed scenarios, based on real-life experiences, are engaging and enable a deeper level of understanding. Previous research has shown that the public’s views can change when exposed to different viewpoints and sources of information [8]. This tool prompts considered views through the presentation of different viewpoints and experiences, while offering users time to reflect on the provided information.

In addition to enabling the provision of considered views to inform policy as an innovative approach, this tool could support a range of activities to inform screening and genomics research, including engagement, consultation, coproduction, and research. The tool and its approach could be applied to other screening scenarios, for example, when public consultation is required, or indeed other scenarios where decision-making needs to be based on a complex set of scientific and experience-based data that may otherwise be hard to access. Future research could include an analysis of tool usage with the potential for interviews with users afterward to explore their understanding and decision-making. This is timely, given the current interest in the use of extended genetic screening techniques to enhance existing newborn screening programs internationally [14,67,68].

### Limitations

Due to project resource constraints, the initial design and development have focused on a web application suitable for access via a PC. The site structure and content require further optimization for viewing on smaller screens or touch-based interaction, as well as consideration of accessibility features to include, for instance, non-English speakers, people with learning differences, and those without access to technologies. In addition to considering mobile access, ongoing development is addressing several recommendations from the testing, including simplification of some of the text, the design of more graphical elements, and the incorporation of voice-over elements.

Through the tool and filmed scenarios, we sought to provide common experiences and emotional responses based on our previous interview findings. However, we recognize that family experiences do vary. We sought to address bias by drawing on previously published research [27] but do acknowledge the potential bias introduced through the researchers’ choice of vignettes and the stakeholders’ lived experiences in reviewing the films and supporting material.

The tool has been developed for consideration of incorporating NGS into the CF newborn screening algorithm. While it is acknowledged that screening programs include many different conditions, it is felt that this work could be used as an exemplar for the development of future tools that could be used to assist parents and professionals with decision-making during the NBS process. The tool is still in development and evaluation. While the process of usability and acceptability testing outcomes are promising, further work is needed, including piloting with parents who are considering CF screening for their child.

### Comparison With Previous Work

As changes are introduced to screening programs to maximize their benefits and reduce their harms, the results produced and how they are interpreted are becoming increasingly complex. The challenge of reaching informed decisions about the nature and content of screening programs is correspondingly also increasing [21]. For parents and stakeholders to understand the implications of introducing expanded newborn screening and extended genetic testing, they need to consider some of the arising ethical questions, including the possible harms (eg, parental anxiety, overdiagnosis, and uncertain results) and the balance of these against potential benefits (eg, early intervention) [10]. These can be complex ideas to communicate to stakeholders and for them to evaluate [10,21,69]. Here, we propose a novel approach to achieving that communication and engagement through using a storytelling approach and scenario-based narratives.

### Conclusions

The online scenario-based tool facilitates access to the considered views of parents and the wider public on genetic testing using storytelling and interactive elements. These views are otherwise difficult to elicit and obtain but are of critical importance to policymakers and stakeholders. Initial feedback on the tool has been positive. Development and further testing continue. It has been identified through the development process that the tool, with its highly interactive nature, will also be of value to those delivering medical training and public health outreach. It allows participants to explore challenging and emotive scenarios in an environment that gives them the opportunity to develop knowledge and empathy. In addition, it has the potential to be used for future research, engagement, consultation, training, outreach, and coproduction. There is also the potential for this sort of online activity to be used as a decision tool for parents deciding whether to have their child screened.

## Abbreviations

CF: cystic fibrosis
CFSPID: CF screen positive, inconclusive diagnosis
CFTR: CF transmembrane conductance regulator
CRMS: CF transmembrane conductance regulator-related metabolic syndrome
HCP: health care professional
NBS: newborn bloodspot screening
NGS: next-generation sequencing
NHS: National Health Service

## Data Availability

Data presented in this study are available on request from the corresponding author. Data are not publicly available due to ethical constraints.
